# Numerical study on vehicle stability under crosswind conditions on desert highways

**DOI:** 10.1038/s41598-025-09286-3

**Published:** 2025-07-02

**Authors:** Zheguang Zhang, Songli Chen, Wei Zhang

**Affiliations:** 1 School of Energy and Transportation Engineering, Inner Mongolia Agricultural University, Hohhot, 010018 China; 2 Inner Mongolia Comprehensive Traffic Operation Monitoring and Emergency Command Center, Hohhot, 010020 China

**Keywords:** Crosswind, Desert highways, Friction coefficient, Numerical simulation, Vehicle stability, Environmental impact, Software, Mechanical engineering

## Abstract

To address the significant impact of strong crosswinds on vehicle driving stability on desert highways, this study employs the aerodynamic six-component force theory and utilizes the vehicle dynamics simulation software CarSim to analyze the stability of small and medium-sized SUVs under varying wind speeds (17.1 m/s, 20.7 m/s, 24.4 m/s, and 28.4 m/s) and vehicle speeds (60 km/h, 80 km/h, 100 km/h, and 120 km/h), with driver operation effects excluded. The results show that both lateral displacement and yaw angle increase significantly with rising wind speed and vehicle speed. Medium-sized SUVs are more susceptible to crosswind effects than small SUVs, exhibiting higher risks of instability and rollover. Although sand accumulation thickness affects the road surface friction coefficient, its influence on lateral vehicle stability is relatively minor. The findings indicate that aerodynamic effects have a much greater impact on vehicle stability than road friction under desert highway conditions. As wind and vehicle speeds increase, the lateral safety offset distance grows while the available reaction time decreases. This study develops a vehicle stability evaluation model under desert crosswind conditions and proposes critical indicators such as the lateral slip risk threshold. The results provide a theoretical basis for the design of highway cross-sections and windproof engineering, as well as a reference for optimizing vehicle active control strategies in desert environments, thereby contributing to improved driving safety on high-speed desert roads.

## Introduction

Desert regions are vast and desolate, accounting for approximately 17% of China’s land area alone^1^. The extreme climatic conditions and desertification pose significant challenges to safe transportation^2–4^. The open and exposed environment in deserts amplifies the effects of crosswinds, making the mitigation of these effects critical for the safe operation of vehicles on desert highways. Crosswind refers to wind blowing perpendicular to the direction of vehicle travel, differing from headwinds and tailwinds. Among various wind directions, crosswinds have the greatest impact on vehicle stability and control, often leading to vehicle instability or rollover^5^. Therefore, investigating the crosswind environment on desert highways and its impact on vehicle safety holds both theoretical and practical significance^6^.

Research on the effects of crosswinds on vehicle safety can be divided into three main aspects: theoretical studies, experimental verification, and numerical simulations. Fu^7^ investigated the relationship between aerodynamic drag coefficients and vehicle shape design through the study of vehicle aerodynamic performance, emphasizing the significant impact of air resistance on vehicle handling, power performance, and fuel economy. The study employed a combination of CFD numerical simulations and wind tunnel experiments to validate the reliability of flow field analysis and proposed a series of optimization measures, including lowering the vehicle front height, optimizing the windshield angle, and improving the smoothness of the underbody structure. The results showed that reasonable shape design could reduce the aerodynamic drag coefficient by 6%, thereby significantly improving fuel efficiency and power performance. The research highlighted the importance of streamlined vehicle design and provided automotive manufacturers with an efficient and cost-effective approach to aerodynamic optimization during the development stage. However, the study offered limited analysis of aerodynamic performance variations under different speed conditions and complex environments, suggesting that future work should incorporate dynamic driving scenarios and higher-precision turbulence models for more in-depth investigation. Zhu et al.^8^ presented wind tunnel test results measuring the aerodynamic coefficients of four types of road vehicles on typical bridge decks, considering various wind directions to observe changes in aerodynamic coefficients with wind angle, aiming to explore the effects of bridge surfaces and different lane positions on vehicle aerodynamic forces.

Many researchers have explored the impact of crosswinds on vehicle stability through full-scale vehicle tests or model wind tunnel experiments. Yang et al.^9^ used CFD and “mosaic” grid techniques to study how crosswinds near sand dunes affect a car’s aerodynamic loads and flow field. They compared three turbulence models: RNG k-ε, LES, and IDDES. The LES model was the most accurate, with a 4.4–7.5% deviation from field data, while RNG k-ε had larger errors (14.8–18.4%). The IDDES model offered a good balance between efficiency and accuracy, though slightly less precise than LES. This study provides valuable insights for evaluating wind-sand effects on vehicle stability in desert areas. Cheli et al.^10^ analyzed the aerodynamic effects on different vehicles under varying scenarios using a 1:10 scale model of heavy vehicles in a wind tunnel, simulating low, medium, and high turbulence. Kee et al.^11^ conducted crosswind occurrence tests, overtaking crosswind tests, and natural road crosswind tests to study high-speed stability under crosswind conditions. Additionally, they developed a crosswind measurement system through wind tunnel tests to understand the aerodynamic and dynamic characteristics of buses subjected to crosswinds. Brandt et al.^12^ used road testing setups to measure relevant wind conditions and identified objective methods for evaluating high-speed stability. They conducted six weeks of field measurements, providing a crucial foundation for developing quantitative standards for high-speed stability assessment. Deng et al.^13^ studied the impact of sand dunes on car driving safety along desert highways, using field monitoring and an improved IDDES method. Field data showed that wind speeds decreased on windward slopes but increased at dune tails. A 3D CFD model was developed to analyze wind-sand flows (WSL), dunes, embankments, and cars. The results showed wind-sand flows significantly affected lateral force, yaw moment, and roll moment, reducing fluctuations by 6.1%, 7.1%, and 6.9%, respectively, at 20 m/s wind speed. The study also highlighted flow separation near dunes and the erosion effect on the car’s body, which could impair visibility and safety. However, it didn’t fully explore tire-road interactions or the effects of particle size and concentration, suggesting areas for further research.

Due to the high safety risks, costs, and environmental factors in real vehicle tests, as well as the scale effects of wind tunnel tests and the difficulty in simulating vehicle dynamic behavior, many researchers have turned to numerical simulations or a combination of model wind tunnel tests and numerical simulations to explore the impact of crosswinds on vehicle safety and stability. Brandt et al.^14^ studied how roof spoiler design affects SUV stability at high speeds under crosswinds, using CFD simulations combined with a vehicle dynamics model. They found that the baseline spoiler caused bistable wake dynamics, leading to significant low-frequency rear lift fluctuations, which impacted steering and stability. The improved spoiler design reduced lift fluctuations, enhanced wake characteristics, and improved overall stability by strengthening tire lateral stiffness. The study highlighted wake instability as the key factor affecting stability, shifting vehicle aerodynamic design focus from load optimization to dynamic response. However, it did not address complex wind fields or multi-dimensional effects, suggesting areas for future research. Bettle et al.^15^ used CFD software to study the effect of truck speed on aerodynamic forces under crosswinds for North American standard-sized transport trucks crossing bridges;. Kwon et al.^16^ used CarSim simulation and wind barrier models to calculate the critical vehicle speed for accidents. Lewington et al.^17^ combined a structured grid CFD model with multibody dynamics simulations to replicate physical testing processes for evaluating vehicle performance under crosswinds. Zhang et al.^18^ adopted a fully coupled vehicle aerodynamics model and two-degree-of-freedom dynamics model, using Matlab and Fluent to analyze the impact of vortex flows on car stability when entering and exiting valleys. Wang B et al.^19^ explored the relationship between aerodynamic drag coefficients and vehicles, discussing the impact of crosswinds on vehicle safety. Taiming et al.^20^ investigated the effect of crosswinds on vehicle aerodynamic stability under different road adhesion coefficients. Mingjin et al.^21^ studied wind field characteristics and vehicle aerodynamic stability on a truss bridge deck under crosswinds, using CFD and wind tunnel experiments to validate simulations. The study found that the main beam’s shielding effect increased wind speed on the windward side, with the attack angle controlling the wind speed profile. The equivalent side wind speed method was deemed superior for assessing lateral stability on wet roads. The study also highlighted the role of vortex characteristics in reducing wind speed, aiding bridge design. However, it mainly focused on static wind fields, with limited exploration of vehicle responses under transient, complex conditions, suggesting areas for further study. Mohebbi’s studies^22–24^ demonstrate that the installation of inclined crosswind barriers, multi-layer wind barriers, and porous wind barriers can effectively reduce the lateral force, lift, and rolling moment acting on high-speed trains under crosswind conditions, thereby significantly enhancing the stability and safety of train operations.

Although several studies have explored the impact of crosswinds on vehicle stability, these studies have primarily focused on specific structural environments, such as bridge decks and tunnel exits, with fewer investigations into the effects of crosswinds in desert road environments. Existing research tends to focus on a limited range of vehicle types, with few studies involving multi-factor analysis under varying combinate -ions of wind speed, vehicle speed, and road surface friction coefficients. Most of the current research centers on lateral displacement as the primary indicator for vehicle stability, overlooking the significant impact of yaw angle on vehicle safety. In fact, yaw angle, as a key parameter for vehicle direction control and dynamic stability, should not be neglected when assessing vehicle safety.

To address the aforementioned limitations, this study employs vehicle dynamics simulation and selects two SUV models of different sizes to investigate the effects of varying crosswind intensities and vehicle speed combinations on lateral vehicle stability (lateral displacement and yaw angle) in desert highway environments. Meanwhile, the influence of sand accumulation on driving safety is simulated by controlling the road surface friction coefficient, further revealing the dominant role of aerodynamic effects under crosswind conditions. This research not only extends the applicability of existing studies to desert highway environments but also provides theoretical support for highway design and vehicle safety operation in desert regions.

## Materials and methods

### System modeling

Carsim provides an intuitive user interface and highly customizable parameter settings, making it well-suited for vehicle modeling requirements. SUVs are the primary choice for vehicles traveling on desert roads because their higher ground clearance, compared to passenger cars, helps prevent the chassis from contacting the sand, reducing the risk of getting stuck and providing greater traction. In this study, the representative small SUV Geely Bin Yue and mid-size SUV Chery Tiggo 8 (2024 model) were selected as research subjects. Their key parameters are shown in Table 1.

**Table 1:** Vehicle technical model Parameters.

Parameter	Compact SUV	Mid-size SUV
Length × Width × Height(mm)	4300 × 1800 × 1609	4700 × 1860 × 1746
Wheelbase(mm)	2600	2710
Front Track/Rear Track(mm)	1551/1600	1582/1604
Frontal Area(m²)	3.7	3.9
Vehicle Weight(kg)	1340	1509
Unsprung Mass(kg)	1139	1283
Tire Model	215/55 R17	235/55 R18
Ixx(kg·m²)	474.1	619.7
Iyy(kg·m²)	2169.5	2858.2
Izz(kg·m²)	2451.2	2975.2
Center of Gravity Height(mm)	642	666

### Basic theory of aerodynamic six-component forces

**Fig. 1:**
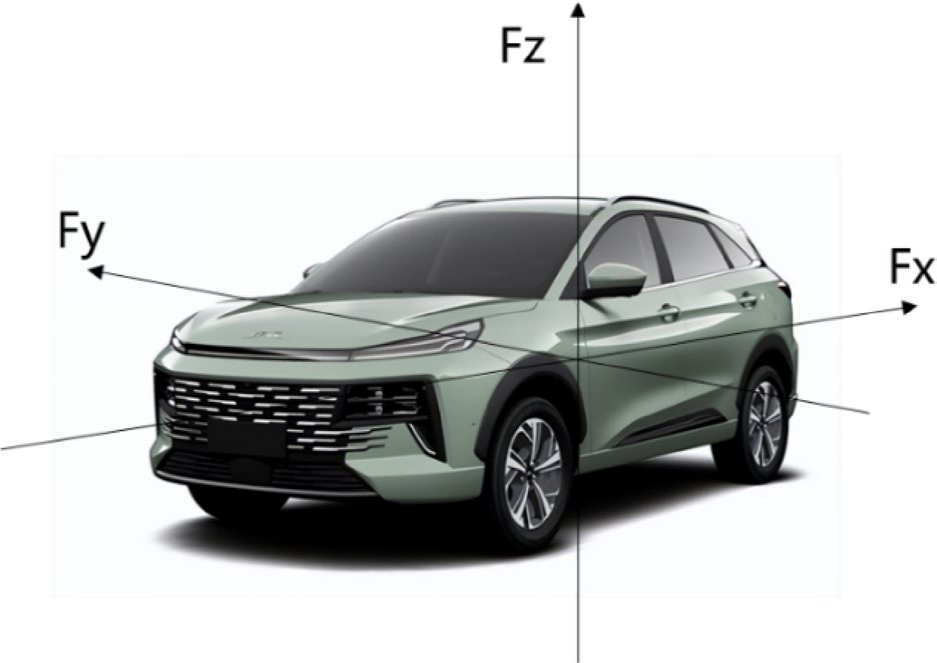
Aerodynamic Six-Component Force Model. (Figure 1 was created by first extracting the car from a photo using the long-press feature on an iPhone, then sending the resulting image via WeChat as a picture, and finally editing it using the PowerPoint software in Office 2024.).

The theory of aerodynamic six-component forces is used to describe the forces and moments acting on a vehicle under aerodynamic influence. It categorizes the aerodynamic effects on a vehicle into three forces (aerodynamic drag, lift, and lateral force) and three moments (yaw moment, pitch moment, and roll moment). This theory is primarily used to study how vehicles respond to wind effects during high-speed driving. These six forces and moments interact with each other, forming the primary aerodynamic factors affecting vehicle performance, as illustrated in Fig. 1.

1 $$\text{Aerodynamic Drag Force Formula}:\:{F}_{X}=\frac{1}{2}A\rho\:{V}_{\infty\:}^{2}{C}_{x}$$

2 $$\text{Aerodynamic Lift Force Formula}:\:{F}_{Z}=\frac{1}{2}\rho\:{V}_{\infty\:}^{2}{C}_{z}$$

3 $$\text{Aerodynamic Side Force Formula}:\:{F}_{Y}=\frac{1}{2}\rho\:{V}_{\infty\:}^{2}{C}_{Y}$$

4 $$\text{Yaw Moment Formula}:\:{M}_{Y}=\frac{1}{2}A\alpha\:\rho\:{V}_{\infty\:}^{2}{C}_{YM}$$

5 $$\text{Pitch Moment Formula}:\:{M}_{P}=\frac{1}{2}A\alpha\:{V}_{\infty\:}^{2}{C}_{PM}$$

6 $$\text{Roll Moment Formula}:\:{M}_{R}=\frac{1}{2}A\alpha\:\rho\:{V}_{\infty\:}^{2}{C}_{RM}$$

In the formulas: $\:{F}_{X}$ represents aerodynamic drag, $\:{F}_{Z}$ represents aerodynamic lift, $\:{F}_{Y}$ represents aerodynamic side force, $\:{M}_{Y}$ represents yaw moment, $\:{M}_{P}$ represents pitch moment, and $\:{M}_{R}$ represents roll moment. $\:{C}_{x}$ is the aerodynamic drag coefficient, $\:{C}_{z}$ is the aerodynamic lift coefficient, $\:{C}_{Y}$ is the aerodynamic side force coefficient. $\:{C}_{YM}$ is the yaw moment coefficient, $\:{C}_{PM}$ is the pitch moment coefficient, and $\:{C}_{RM}$ is the roll moment coefficient. All these coefficients can be found as curve plots within the Carsim software.

### Model construction and simulation execution

#### Wind speed settings

In the CarSim software, wind speed is set as a constant wind and expressed in km/h. To comprehensively study the effects of different wind speeds and vehicle speeds on vehicle response to crosswinds, this paper selects crosswind speeds of 17.1 m/s, 20.7 m/s, 24.4 m/s, and 28.4 m/s, which correspond to the maximum wind speeds of levels 7, 8, 9, and 10 in the Chinese “Wind Force Classification Standard.” These values represent typical natural crosswind intensities and cover the range from strong winds to violent winds commonly observed in typical desert highway regions in China, such as the Taklamakan and Badain Jaran deserts, thus ensuring representativeness and engineering applicability.

The aerodynamic coefficients of the vehicle used in this study are based on the built-in parameters of a typical medium-sized passenger car model in the CarSim software, which are primarily calibrated according to real vehicle experiments and wind tunnel test data. This model has been validated and applied in multiple studies on vehicle safety under crosswind conditions, demonstrating strong engineering representativeness^25–27^. Since the focus of this research is on analyzing the relative influence trends of external disturbances such as wind speed, vehicle speed, and sand accumulation, this generic aerodynamic model is employed to ensure the general comparability of simulation results. Future work will further integrate CFD simulations and wind tunnel tests to independently analyze and validate the aerodynamic parameters, thereby improving model accuracy.

When a vehicle is subjected to crosswind, the resultant forces of drag, lift, and lateral force act at the center of pressure. In previous studies, the aerodynamic reference height for the center of pressure is typically selected between 1.0 m and 1.5 m. In this study, the center of pressure is set at a height of 1.2 m. The air density is 1.206 kg/m³.

#### Driving mode settings

**Fig. 2:**
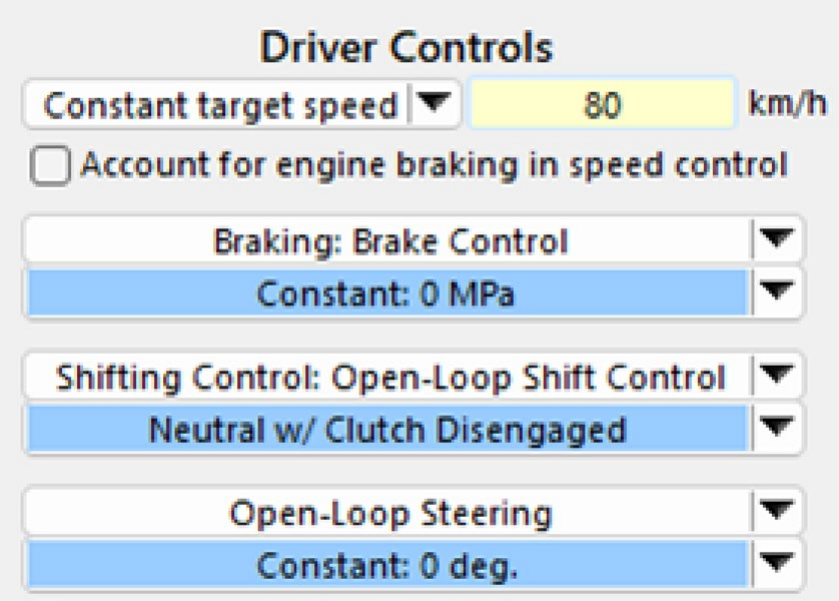
Driving Mode Settings.

As shown in Fig. 2, this study adopts the open-loop driving control mode in CarSim to exclude the influence of driver operations on vehicle dynamic response, thereby more realistically simulating the passive response state of vehicles under strong crosswind and sand accumulation disturbances on desert highways. This approach is particularly suitable for evaluating critical instability conditions. In this mode, the vehicle maintains no throttle, no braking, a zero steering wheel angle, and the transmission is set to neutral, ensuring that external disturbances are the dominant influencing factors. Vehicle speeds are selected within the range of 60–120 km/h, based on speed limits stipulated by the Road Traffic Safety Law and the Highway Route Design Specifications issued by the Ministry of Transport, representing common operating speeds on highways. Typical conditions are set at 20 km/h intervals to analyze the coupled effects of wind speed and vehicle speed, providing good comparability and observable trend analysis. Additionally, this study focuses primarily on passive response characteristics without driver intervention. Future research will incorporate driver models and vehicle stability control systems (e.g., ESC) to compare their regulatory effects on instability boundaries, enabling a more comprehensive assessment of driving safety on desert highways.

#### Road settings

**Fig. 3:**
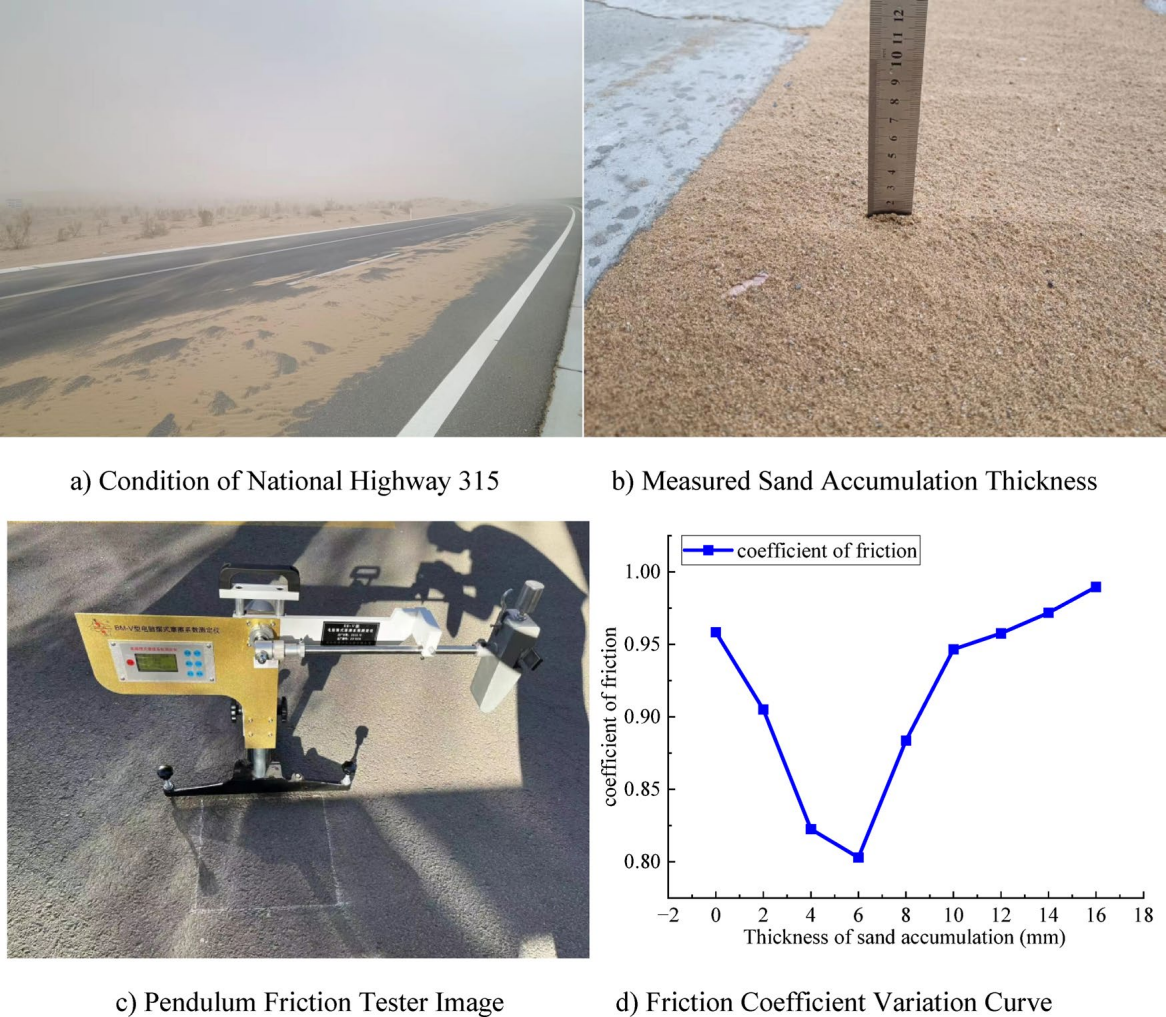
Relationship between Sand Accumulation Thickness and Friction Coefficient.

In this study, field tests were conducted to collect data on sand accumulation thickness on desert highways. According to the measured data, sand accumulation thickness is closely related to desert crosswinds. Strong winds carry sand particles onto the road surface, resulting in the formation of sand layers. To further quantify this process, a pendulum-type friction tester was used to measure the friction coefficient of the sand layer. The relationship between sand accumulation thickness and friction coefficient is shown in Fig. 3.

According to the results shown in Fig. 3, the relationship between sand accumulation thickness and road surface friction coefficient exhibits a trend of first decreasing and then increasing, with the later changes tending to stabilize. The friction coefficient reaches its minimum at a sand accumulation thickness of 6 mm. Therefore, in the simulation, the road segment is set as a straight line, and two surface conditions—bare road and road with 8 mm sand accumulation thickness—are selected as control groups, with friction coefficients of 0.9584 and 0.8836, respectively. This setup will help better analyze the effect of sand accumulation thickness on the friction coefficient and provide data support for further research.

#### Accuracy evaluation

**Fig. 4:**
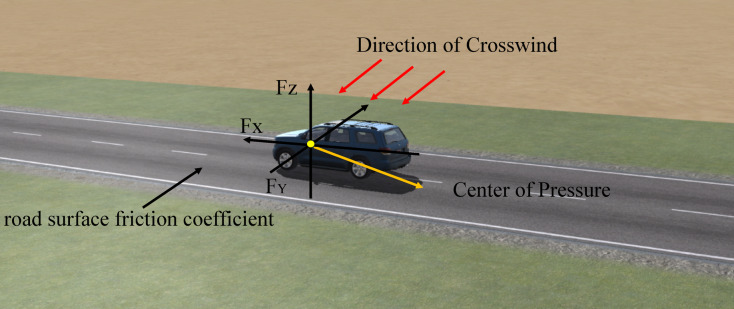
Schematic of a vehicle passing through a desert highway under the effect of crosswinds. (Figure 4 illustrates the ‘Video’ function in CarSim software, which presents simulated scenarios under different conditions. Screenshots were taken and edited in PowerPoint (Office 2024), where the sand-covered areas on the road were highlighted with irregular shapes and filled using the Eyedropper tool by sampling colors from the surrounding desert.)

Figure 4 illustrates the influence of crosswind on a vehicle traveling on a desert highway. Lateral displacement and yaw angle are adopted as key metrics to evaluate driving stability and safety.

Lateral displacement refers to how much a vehicle moves off its original path due to crosswinds. A larger lateral displacement means the vehicle is more affected by crosswinds, which reduces stability and makes it harder to control. This increases the risk of collisions or running off the road, and drivers may need to make frequent steering adjustments, leading to fatigue and pressure. SUVs, with higher ground clearance, tend to experience larger lateral displacements and are more prone to instability or rollover.

Yaw angle is the angle at which a vehicle’s longitudinal axis deviates from its travel direction due to crosswinds. A larger yaw angle indicates greater deviation, requiring the driver to adjust the steering to correct the course. Increased yaw angles worsen vehicle stability, especially at higher wind speeds and angles, and can lead to mental fatigue from constant steering adjustments. SUVs are particularly vulnerable to rollover risks due to their higher ground clearance and larger yaw angles.In summary, under the influence of crosswinds, an increase in lateral displacement and yaw angle weakens the vehicle’s straight-line stability and controllability. A higher lateral displacement increases the risk of the vehicle drifting out of its lane, while an increase in yaw angle makes it more difficult for the vehicle to maintain its intended direction of travel. The combined effect of both significantly elevates the risk of loss of control and accidents. Therefore, studying the trends and impacts of lateral displacement and yaw angle can provide valuable reference data for vehicle design and driving safety under crosswind conditions.

## Simulation results and analysis

According to previous research results, a wind direction angle of 120° has the greatest impact on vehicle stability; therefore, a uniform wind direction angle of 120° is adopted to highlight the distinctive characteristics of crosswind on desert highways compared to other roads. The reference indicators are the vehicle’s lateral displacement and yaw angle. The data sampling interval is 0.5 s, and the simulation duration is 10 s.

### The impact of different wind speeds and sand accumulation on vehicle stability

**Fig. 5:**
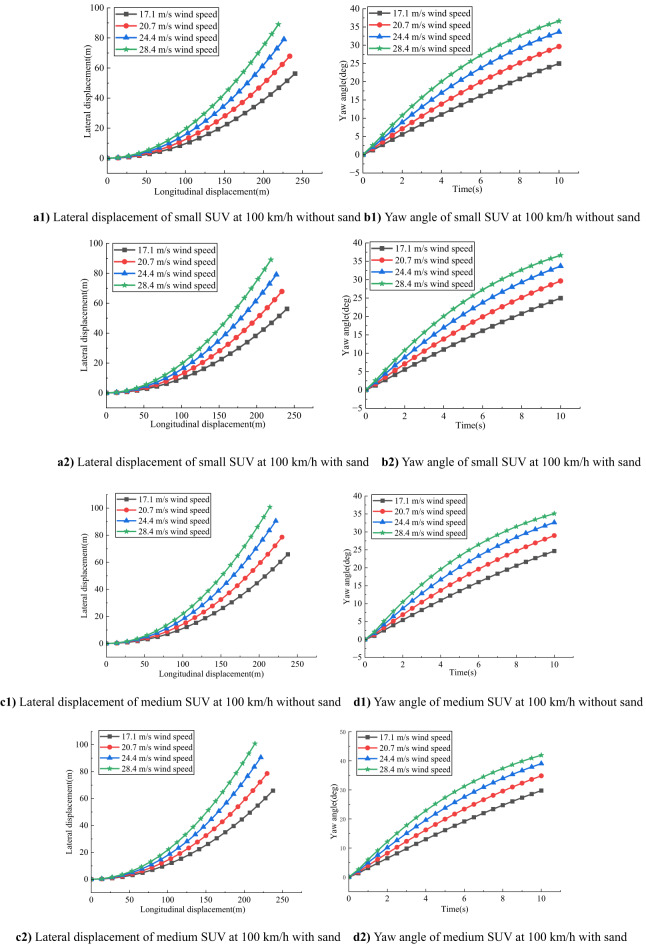
Impact of crosswind at different wind speeds on two vehicle types at 100 km/h under sanded and non-sanded conditions.

As shown in Fig. 5, for both small and medium-sized SUVs traveling at a speed of 100 km/h, the lateral displacement and yaw angle increase significantly with higher crosswind speeds, indicating that stronger winds have a greater impact on vehicle stability. Specifically, under non-sand-covered road conditions, the small SUV exhibits a lateral displacement ranging from 56.2800 m to 89.0262 m, and a yaw angle ranging from 24.9870° to 31.6156°. When sand is present on the road, the lateral displacement changes only slightly (56.2799 m to 89.1022 m), but the yaw angle increases notably, reaching a maximum of 36.6393°. This suggests that while sand accumulation has limited influence on lateral displacement, it amplifies the vehicle’s yaw response.

In comparison, the medium-sized SUV shows greater overall lateral displacement and yaw angle under the same conditions. Without sand accumulation, its lateral displacement ranges from 65.8833 m to 100.7748 m, and its yaw angle from 29.7547° to 41.9249°. With sand, the lateral displacement remains similar, but the yaw angle approaches the upper bound. These results indicate that larger vehicles, due to their greater surface area exposed to wind, are more susceptible to lateral instability and require enhanced stability control measures.

Moreover, the comparison between sand-covered and non-sand-covered conditions reveals that while reduced road friction caused by sand has a relatively minor effect on lateral displacement, it significantly exacerbates yaw behavior—especially under strong wind conditions—potentially leading to severe steering failure. Therefore, in addition to wind protection structures, desert highways should also prioritize sand removal and pavement maintenance to prevent compounding effects from both wind and sand on driving safety.

At a vehicle speed of 100 km/h, both the lateral displacement and yaw angle of compact and mid-size SUVs increase with the intensification of crosswind. Under sand-covered road conditions, these indicators are consistently higher than those observed on clean pavement, indicating that surface sand further undermines lateral vehicle stability.

From the perspective of aerodynamic six-component forces, the increase in crosswind speed significantly amplifies the lateral aerodynamic force and yawing moment acting on the vehicle. The enhanced lateral force causes the vehicle to deviate from its intended path, resulting in a lateral displacement that grows approximately in proportion to the square of wind speed. Meanwhile, the yawing moment increases as wind speed rises, particularly due to asymmetric loading on the front and rear sections of the vehicle. These aerodynamic effects substantially degrade lateral stability under high wind conditions, further amplifying lateral displacement and yaw angle responses.

A comparative analysis between the two vehicle types reveals that although the trends are similar, the mid-size SUV exhibits significantly greater lateral displacement and yaw angle than the compact SUV across all wind conditions. While a heavier vehicle is theoretically expected to offer better resistance to wind disturbances due to increased inertia, the mid-size SUV’s larger frontal area and higher center of gravity make it more susceptible to aerodynamic forces. This finding highlights that the frontal area and center of gravity height play a more dominant role than vehicle mass in determining a vehicle’s crosswind stability, especially under desert highway conditions.

### Impact of different speeds and sand accumulation on vehicle stability

**Fig. 6:**
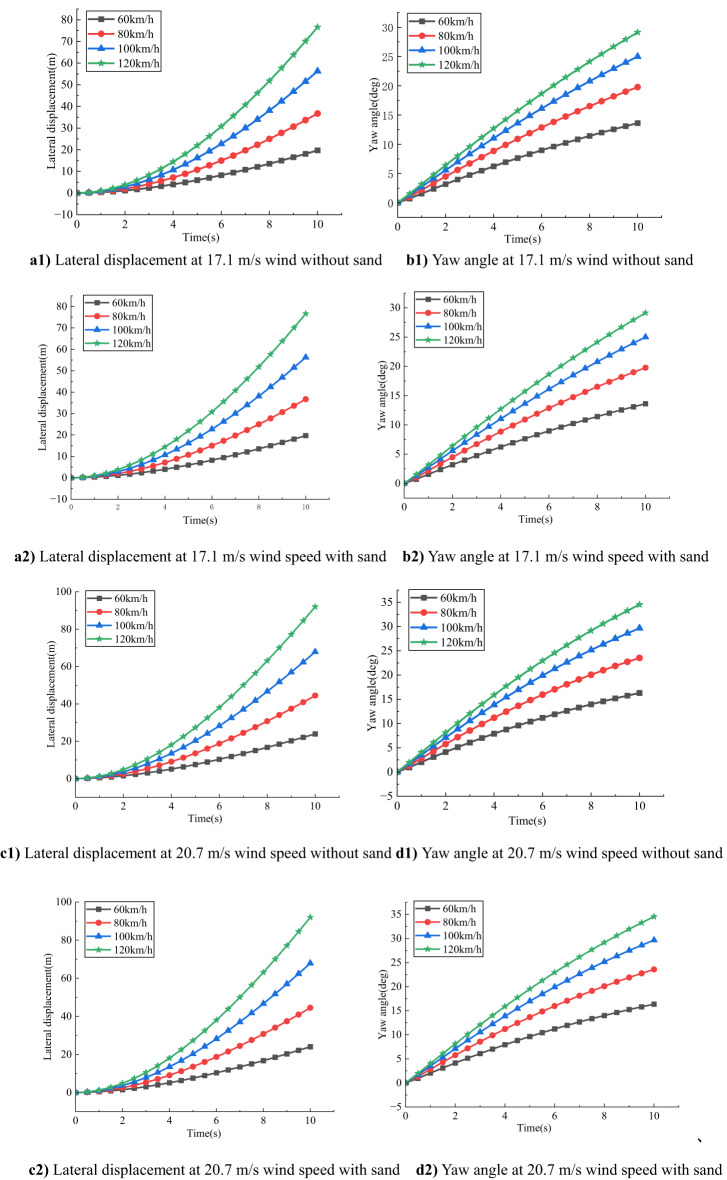

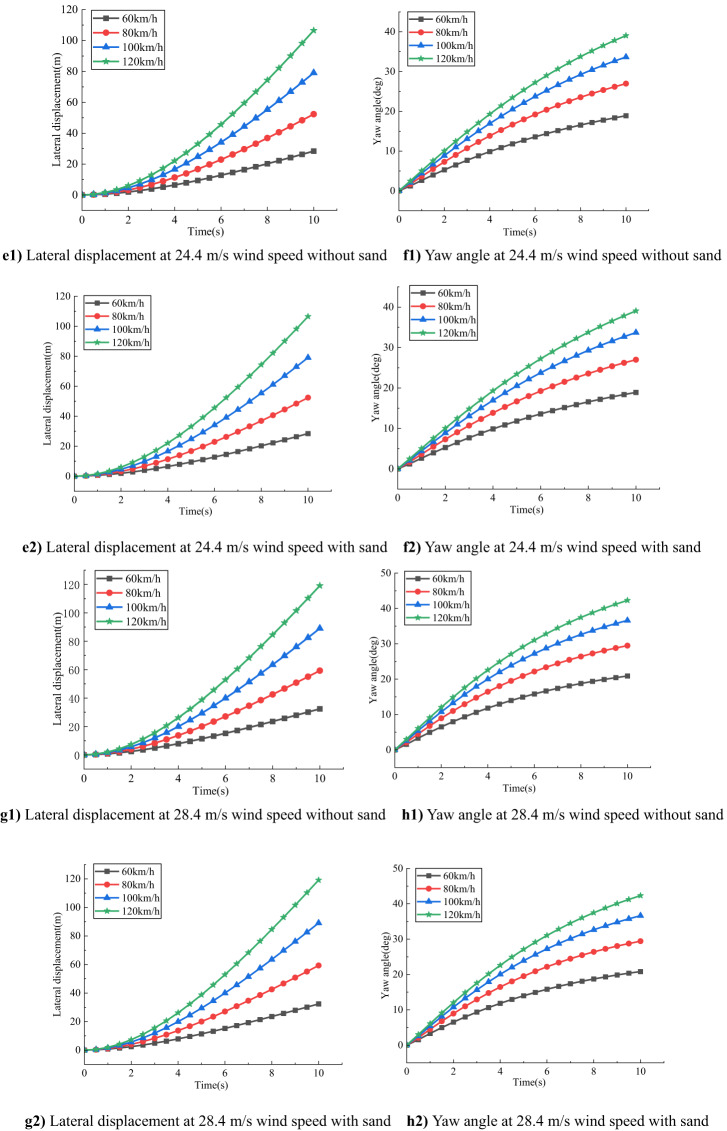
Comparison of the effect of different speeds on a small SUV at the same wind speed.

As shown in Fig. 6, for compact SUVs under the same crosswind speed, both lateral displacement and yaw angle increase significantly with vehicle speed. This indicates that higher speeds amplify the destabilizing effects of crosswinds, reducing overall driving stability.

At a wind speed of 17.1 m/s, without sand accumulation, the lateral displacement and yaw angle are 19.7133 m and 13.6113° respectively at 60 km/h, increasing to 76.6170 m and 29.1278° at 120 km/h. Under sand-covered road conditions, these values increase slightly, suggesting that at moderate wind speeds, the effect of sand accumulation on vehicle response is relatively limited.

When the wind speed rises to 20.7 m/s, the lateral displacement and yaw angle further increase to 23.9681 m and 16.3001° at 60 km/h, and to 91.9849 m and 34.5245° at 120 km/h under no-sand conditions. With sand accumulation, both parameters exhibit slightly higher values, indicating that the effect of crosswind is more pronounced at this level, and sand accumulation begins to play a more noticeable role.

At a wind speed of 24.4 m/s, the vehicle response intensifies significantly. Under no-sand conditions at 120 km/h, the lateral displacement reaches 106.5450 m, and the yaw angle is 39.0359°. Under sand-covered conditions, both values slightly increase, indicating that sand accumulation further exacerbates the vehicle’s instability at high wind speeds.

When the wind speed reaches 28.4 m/s, the vehicle experiences the most severe deviations. At 120 km/h under no-sand conditions, the lateral displacement and yaw angle are 119.0981 m and 42.2862°, respectively. With sand accumulation, these values rise slightly to 119.1663 m and 42.3119°, underscoring the critical threat posed by high-speed crosswind driving on sand-laden roads.

In summary, both lateral displacement and yaw angle increase with wind speed and vehicle speed, especially under high wind and high-speed conditions. Although the effect of sand accumulation is negligible under low to moderate wind speeds, it becomes increasingly significant under strong crosswinds. Therefore, reducing vehicle speed is essential for ensuring driving safety on desert highways prone to sand accumulation and intense crosswinds.

**Fig. 7:**
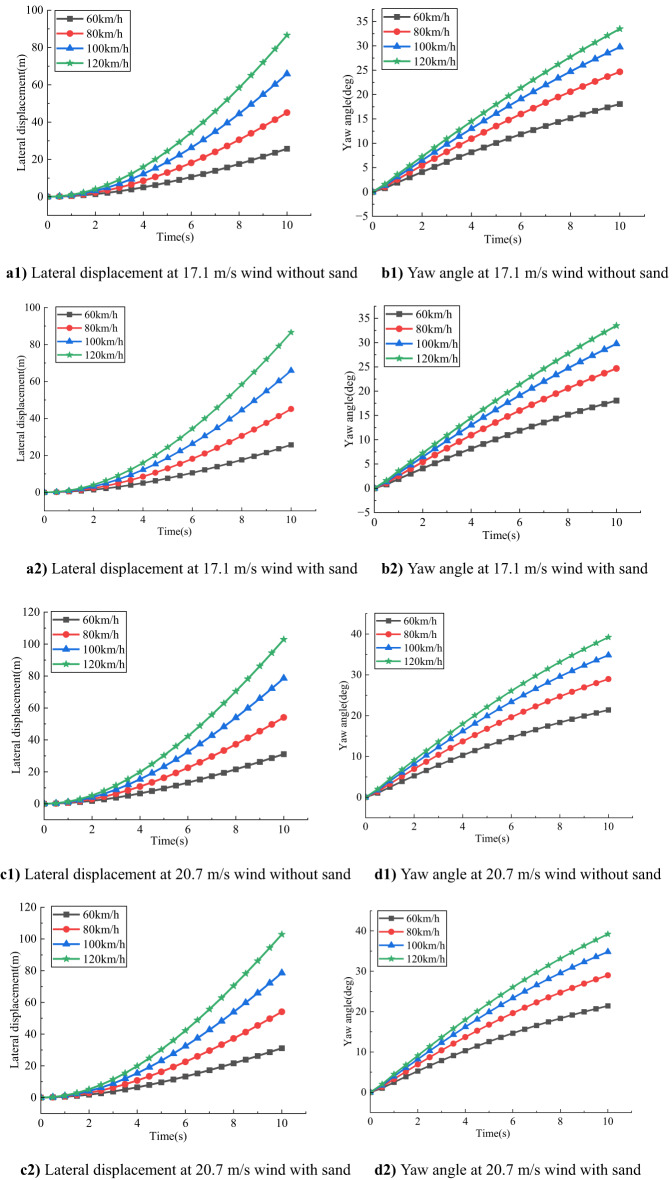

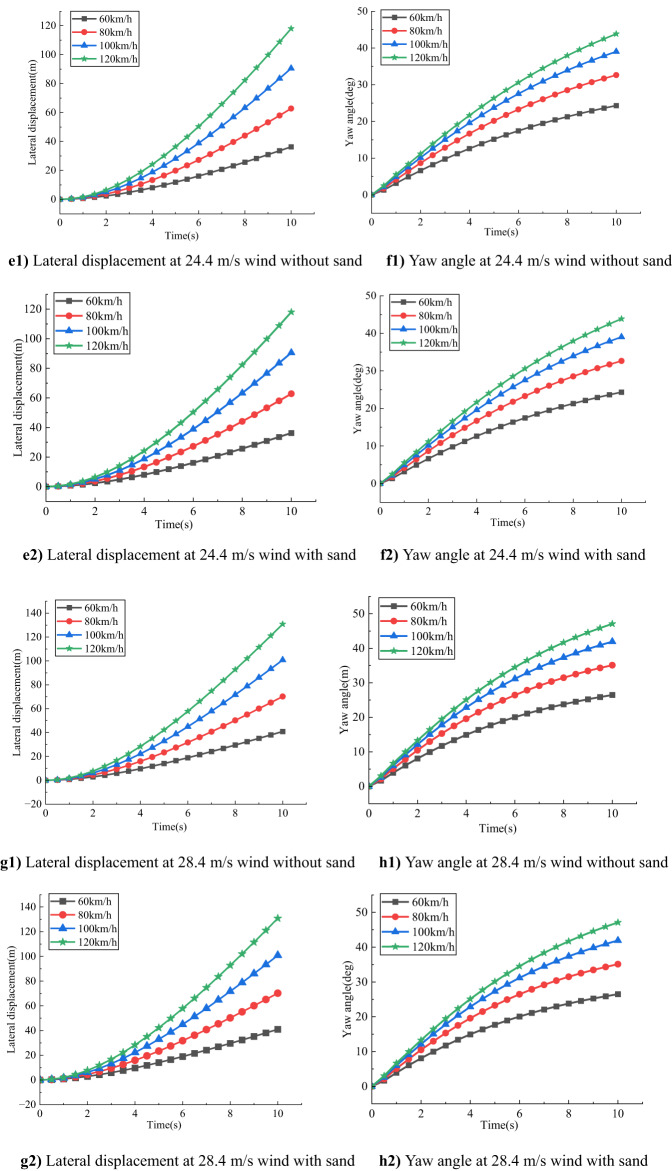
Comparison of the impact of different speeds on a medium SUV under the same wind speed.

As shown in Fig. 7, under the same wind speed conditions, the lateral displacement and yaw angle of the mid-size SUV both significantly increase with increasing vehicle speed, indicating a combined weakening effect of wind speed and vehicle speed on the vehicle’s lateral stability.

Specifically, at a wind speed of 17.1 m/s, under the no-sand accumulation condition, when the vehicle speed increases from 60 km/h to 120 km/h, the lateral displacement rises from 25.7481 m to 86.5768 m, and the yaw angle increases from 18.0632° to 33.4956°. Under the sand accumulation condition, the corresponding values change slightly from 25.7491 m to 86.5768 m and from 18.0651° to 33.4956°, indicating that the effect of sand accumulation is negligible at low wind speeds.

When the wind speed increases to 20.7 m/s, the vehicle’s response to wind loads is further enhanced. In the absence of sand accumulation, the lateral displacement and yaw angle at low speed (60 km/h) are 31.0901 m and 21.4014°, respectively, while at high speed (120 km/h) they reach 102.8914 m and 39.2107°. Similar trends are observed under sand accumulation conditions, suggesting that sand slightly increases aerodynamic disturbances around the vehicle, leading to more pronounced yaw and lateral displacement.

At a higher wind speed of 24.4 m/s, lateral stability deteriorates markedly. Without sand, the lateral displacement and yaw angle at low speed are 36.2804 m and 24.3165°, respectively, while at 120 km/h, these values increase to 118.0173 m and 43.8417°. Under sand accumulation conditions, the corresponding values are slightly higher at 36.3003 m and 118.0543 m, with the yaw angle increasing marginally to 43.8516°. This minor but persistent increase suggests that the combined effect of sand and wind speed near critical wind velocities can create a significantly unstable aerodynamic environment.

At an extreme wind speed of 28.4 m/s, the vehicle exhibits the strongest response. At 60 km/h, the lateral displacement and yaw angle without sand accumulation are 40.8941 m and 24.4699°, respectively; at 120 km/h, these values peak at 130.7719 m and 47.0841°. Under sand accumulation conditions, these parameters increase slightly to 130.7971 m and 47.0969°, while the yaw angle at low speed even surges to 26.9408°, indicating that sand accumulation has a more pronounced disturbing effect on the vehicle’s aerodynamic characteristics under strong crosswind conditions.

Overall, the mid-size SUV demonstrates significant sensitivity to both vehicle speed and wind load under strong crosswind conditions, with lateral displacement and yaw angle increasing rapidly with wind speed and vehicle speed. Although the influence of sand accumulation on vehicle behavior is less pronounced at high speeds, its contribution to yaw angle at lower speeds should not be overlooked.

Based on Figs. 6 and 7, this study compares the lateral displacement and yaw angle of two different vehicle types, namely compact SUVs and midsize SUVs, as single variables under various crosswind speeds. The results show that, under the same crosswind speed, both vehicle types exhibit a significant increase in lateral displacement and yaw angle as vehicle speed increases. Moreover, these values are larger when sand accumulates on the road surface.

From the perspective of aerodynamic dynamics, lateral aerodynamic force is proportional to the square of the vehicle speed. Although the crosswind intensity remains constant, the aerodynamic lateral force acting on the vehicle increases rapidly with speed, directly pushing the vehicle toward the crosswind direction, thereby increasing lateral displacement. Under crosswind conditions, the pressure difference on the windward side generates a yaw moment about the vehicle’s center of gravity, which also increases with vehicle speed, leading to greater yaw angle.

Additionally, as vehicle speed increases, the relative wind speed (the resultant of the vehicle’s travel speed and crosswind speed) changes, causing a reduction in the effective crosswind incident angle. However, the aerodynamic effects remain enhanced, making the vehicle more prone to yawing and lateral deviation at higher speeds.

Comparing the two vehicle types, midsize SUVs consistently show larger lateral displacements and yaw angles than compact SUVs under the same conditions. This indicates that due to their larger frontal area and higher center of gravity, midsize SUVs generate greater yaw moments. Although midsize SUVs are heavier, the aerodynamic effects under high-speed crosswind conditions have a more significant impact on their stability than vehicle weight.

### Effect of different sand deposits on vehicle stability

As shown in Tables 2 and 3, with the decrease of the road surface friction coefficient, the lateral displacement and yaw angle of both the small SUV and the medium SUV increase accordingly; however, the magnitude of these changes is not significant. For the small SUV, under the conditions of 100 km/h vehicle speed and level 10 wind, when the sand accumulation thickness increases from 0 mm to 8 mm, the lateral displacement changes from 89.0262 m to 89.1022 m, and the corresponding yaw angles are 36.6156° and 36.6393°, respectively. The maximum difference in lateral displacement is 0.139 m, and the maximum difference in yaw angle is 0.0297°, with variations controlled within 0.2%.

Similarly, for the medium SUV under the same conditions, the lateral displacements are 130.7719 m, 130.8279 m, and 130.7971 m, while the yaw angles are 47.0841°, 47.1113°, and 47.0970°, respectively. The maximum difference in lateral displacement is 0.056 m, and the maximum difference in yaw angle is 0.0272°, indicating relatively small variation.

**Table 2:** Influence of different sand thickness on the safety of small SUV.

vehicle speed(km/h)	Wind speed	Lateral displacement(m)		Yaw angle(deg)	
		0 mm	8 mm	0 mm	8 mm
60 km/h	17.1 m/s	19.7139	19.7139	13.6113	13.6113
	20.7 m/s	23.9681	24.0483	16.3008	16.3524
	24.4 m/s	28.4341	28.4685	18.8923	18.9153
	28.4 m/s	32.4111	32.397	20.9067	20.8302
80 km/h	17.1 m/s	36.7322	36.7322	19.7694	19.7694
	20.7 m/s	44.4859	44.5219	23.5341	23.5673
	24.4 m/s	52.3451	52.4266	26.9548	26.99
	28.4 m/s	59.2883	59.3066	29.4772	29.4239
100 km/h	17.1 m/s	56.28	56.28	24.987	24.987
	20.7 m/s	67.8639	67.8646	29.6529	29.6531
	24.4 m/s	79.0674	79.1516	33.659	33.6959
	28.4 m/s	89.0262	89.1022	36.6156	36.6393
120 km/h	17.1 m/s	76.617	76.617	29.1278	29.1278
	20.7 m/s	91.985	91.9843	34.5245	34.5244
	24.4 m/s	106.545	106.5685	39.0359	39.056
	28.4 m/s	119.0981	119.1663	42.2862	42.3119

**Table 3:** Influence of different sand thickness on the safety of Mid-Size SUV.

vehicle speed(km/h)	Wind speed	Lateral displacement(m)		Yaw angle(deg)	
		0 mm	8 mm	0 mm	8 mm
60 km/h	17.1 m/s	25.7481	25.7491	18.0632	18.0651
	20.7 m/s	31.0901	31.1306	21.4014	21.4259
	24.4 m/s	36.2804	36.3003	24.3165	24.3265
	28.4 m/s	40.8941	40.9554	26.4699	26.4908
80 km/h	17.1 m/s	45.1091	45.1091	24.6716	24.6716
	20.7 m/s	54.0703	54.1078	28.9738	29.0006
	24.4 m/s	62.7609	62.8043	32.6277	32.6474
	28.4 m/s	70.1769	70.2188	35.1026	35.114
100 km/h	17.1 m/s	65.8833	65.8833	29.7547	29.7547
	20.7 m/s	78.5903	78.5912	34.826	34.8267
	24.4 m/s	90.5405	90.5746	39.0126	39.0308
	28.4 m/s	100.7748	100.8108	41.9249	41.9182
120 km/h	17.1 m/s	86.5768	86.5768	33.4956	33.4956
	20.7 m/s	102.8914	102.8914	39.2107	39.2107
	24.4 m/s	118.0173	118.0243	43.8417	43.8516
	28.4 m/s	130.7719	130.7971	47.0841	47.097

This indicates that although changes in the road surface friction coefficient significantly affect the longitudinal dynamic performance of the vehicle (such as braking and acceleration), their impact on lateral displacement and yaw angle under crosswind conditions is minimal. Under crosswind conditions, the vehicle’s lateral displacement and yaw are mainly influenced by aerodynamic forces. Particularly at high speeds, aerodynamic effects become the dominant factor affecting lateral stability. In contrast, variations in the road surface friction coefficient have a relatively weak influence on lateral movement and yaw moment caused by crosswinds.

## Evaluation method

### Calculation formula for lateral skid danger threshold

**Fig. 8:**
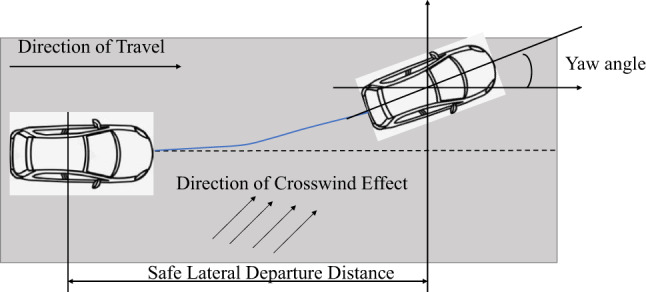
The Impact of Crosswind on Vehicle Stability. (Figure 8 was also created using PowerPoint in Office 2024.).

When a vehicle is driving at high speed on the road, it may experience lateral displacement, potentially leading to traffic accidents, due to sudden events or external factors such as crosswinds. To determine the dangerous critical conditions of lateral displacement, the position where the vehicle’s front deviates from the lane line is defined as the dangerous critical state, and the distance between the vehicle’s center of mass and the road centerline at this point is defined as the lateral skid danger threshold. As shown in Fig. 8, the lateral skid danger threshold varies with changes in vehicle size parameters, driving conditions, and crosswind environments.

7 $$L_D=\frac{L_w}{2}-d_v sin\theta-\frac{W_v}{2}cos\theta$$

8 $${\text{T=}}{{\text{T}}_d}{\text{-}}{{\text{T}}_s}$$

9 $${\text{L=}}{{\text{L}}_d}{\text{-}}{{\text{L}}_s}$$

In the formula: ${{\text{L}}_D}$ represents the lateral deviation danger threshold, m;; ${{\text{L}}_w}$ represents the lane width, m; ${{\text{d}}_v}$ represents the vertical distance from the center of mass to the front of the vehicle, m; ${{\text{W}}_v}$ represents the vehicle width, m; T represents the safety reaction time, s; ${{\text{T}}_d}$ represents the time at which the vehicle deviates such that its front end touches the lane line, s; ${{\text{T}}_s}$ represents the time at which the vehicle begins to drift laterally under the action of crosswind, s;${{\text{L}}_d}$ represents the distance traveled by the vehicle when the front of the vehicle reaches the lane line during lateral deviation, m; ${{\text{L}}_s}$ represents the distance traveled by the vehicle from the onset of lateral deviation under crosswind conditions, m. θ represents the vehicle yaw angle.

### Lateral deviation safety distance and safety response time

**Fig. 9:**
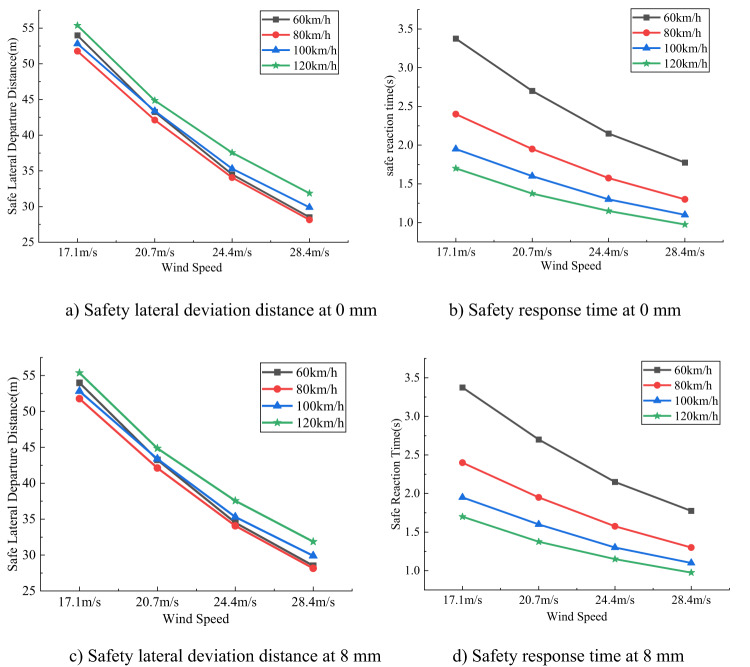
Lateral deviation distance and response time of small SUV under different sand thickness conditions.

**Fig. 10:**
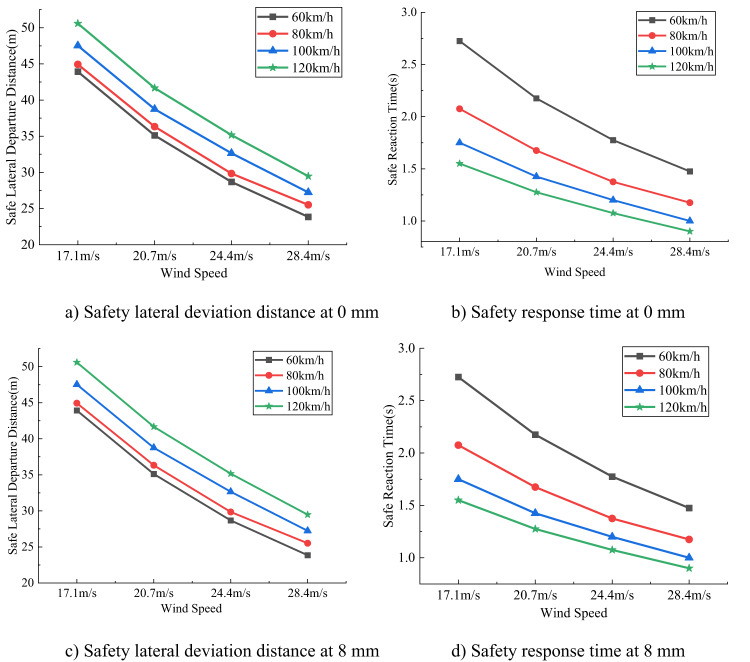
Lateral deviation distance and response time of medium SUV under different sand thickness conditions.

As shown in Figs. 9 and 10, for SUVs traveling at the same speed, the lateral deviation safety distance and safety response time decrease as wind speed increases. At the same wind speed, higher vehicle speeds result in larger lateral deviation safety distances but shorter safety response times. However, the thickness of sand accumulation has minimal impact on the changes in lateral deviation safety distance and safety response time. Although the trends are similar for medium SUVs and small SUVs, the medium SUV exhibits larger lateral deviation safety distances and shorter safety response times.

Comprehensive analysis shows that wind speed and vehicle speed are the primary factors affecting the vehicle’s side-slip safety distance and safe reaction time. As wind speed increases, the lateral force and yaw moment acting on the vehicle strengthen, leading to a greater tendency to deviate from the original driving trajectory, which reduces the side-slip safety distance and shortens the driver’s safe reaction time. Although higher vehicle speed, due to greater momentum, can slightly improve the side-slip safety distance by resisting short-term disturbances, the shortened travel time per unit distance requires the driver to correct more quickly, still significantly shortening the reaction time. Compared to small SUVs, mid-size SUVs have larger side-slip safety distances because of greater mass and wider track, but their higher center of gravity and stronger yaw response result in shorter reaction times. In contrast, thin sand deposits have little effect on the vehicle’s aerodynamic characteristics and tire grip, thus their impact on side-slip safety distance and safe reaction time is minimal.

## Discussion and conclusion

### Research summary

This study systematically investigated the effects of crosswinds on the driving stability of small and medium SUVs in desert highway environments through vehicle dynamics simulation. It focused on analyzing the roles of key variables such as wind speed, vehicle speed, and road surface friction coefficient, highlighting the dominant role of aerodynamic effects under crosswind conditions. The study also quantified lateral deviation safety distance and safety reaction time. The main conclusions are as follows:

1. Wind Speed and Vehicle Lateral Stability.
2. As wind speed increases, the lateral displacement and yaw angle of vehicles significantly rise, with medium SUVs being more noticeably affected by crosswinds compared to small SUVs. This indicates that when designing SUVs for desert highways, priority should be given to optimizing aerodynamic features and reducing frontal area to enhance the vehicle’s resistance to crosswinds.
3. Vehicle Speed and Aerodynamic Effects.
4. The lateral force on a vehicle is proportional to the square of its speed, indicating that the impact of aerodynamic effects on lateral stability becomes more pronounced at higher speeds. This finding further underscores the importance of establishing reasonable speed limits on desert highways, particularly during strong wind conditions. It is recommended to enforce strict speed controls in areas with high wind speeds to reduce safety risks caused by crosswinds.
5. Impact of Road Surface Friction Coefficient.
6. Although sand accumulation on desert highways can reduce the road surface friction coefficient, its effect on vehicle lateral displacement and yaw angle is relatively minor. Under high wind conditions, aerodynamic effects become the dominant factor. Therefore, to enhance driving safety on desert highways, efforts should focus on improving vehicle structural design and aerodynamic performance rather than solely relying on enhancing road surface friction characteristics.
7. Lateral Safety Distance and Safety Reaction Time.
8. The study found that lateral safety distance increases with vehicle speed but decreases with increasing wind speed, while safety reaction time decreases with both higher vehicle speed and stronger wind speed. This result highlights the safe operating range of vehicles and the reaction limits of drivers under varying speed and wind conditions. It underscores the importance of dynamically monitoring crosswind environments and adjusting driving strategies in real-time based on vehicle parameters.

In summary, wind speed and vehicle speed are the dominant factors affecting lateral stability on desert highways. Optimizing vehicle aerodynamic performance and scientifically setting regional speed limits are key strategies for improving driving safety in desert environments. Furthermore, the lateral stability evaluation index system and risk threshold assessment method proposed in this study provide quantitative support for engineering designs such as speed limit setting, windbreak (or shelterbelt) placement, and slope width reservation in desert highway projects. They also offer theoretical references for the development of stability control algorithms for small and medium-sized vehicles.

Although this study was conducted with a typical desert highway in western China as the background for model construction and simulation verification, the proposed analytical method and evaluation framework have good general applicability and are also suitable for similar research and road engineering design in typical desert regions such as the Middle East, North Africa, and the western United States.

### Future work

To further enhance the practical application and engineering feasibility of this study, future research will focus on the following aspects:

1. Establishing a scaled physical experiment platform to conduct validation tests of vehicle responses under wind disturbances;
2. Integrating wind speed sensors and vehicle attitude detection devices to enable dynamic data collection in desert wind disturbance scenarios;
3. Designing active wind disturbance identification and simplified control logic, and verifying its adaptability in advanced driver-assistance systems (ADAS) such as ESC and active steering;
4. Introducing human driver behavior models to explore the influence of driver interventions on vehicle dynamic responses, thereby supporting the development of driver-assistance strategies.

## Data Availability

The datasets generated and analyzed during the current study are available from the corresponding author on reasonable request.
